# Current Challenges for German Business in the Russian Market

**DOI:** 10.1134/S1019331622100094

**Published:** 2022-09-22

**Authors:** A. V. Kotov

**Affiliations:** Institute of Europe, Russian Academy of Sciences, Moscow, Russia

**Keywords:** Russia, Germany, European Union, sanctions, trade and economic cooperation, decision strategy, German business, external administration

## Abstract

The reaction of leading German companies in the Russian market after the start of the special military operation on the territory of Ukraine is considered. Companies interested in resolving the situation as soon as possible decided whether to leave the Russian market or not. The author analyzed the decisions of firms in the general context of winding down German business activities after 2014. After the acute phase of the pandemic ended, trade and economic cooperation between Russia and Germany showed a tendency to recover. However, since February 24, 2022, German companies with a small number of exceptions have generally supported anti-Russian sanctions. The conceptual typology of firms’ decisions, i.e., exit, activity compression, stabilization, and continuation of work in a difficult environment, is explored. It is concluded that the long-term trajectory of the real actions of German companies has fundamentally changed. The analysis made it possible to establish that the foundation of pragmatic market relations towards Russia is being eroded in the German foreign economic policy. This explains why German companies declare their willingness to pay. If during the annexation of Crimea to Russia groups of lobbyists for the interests of the German economy fought to maintain contacts, now such efforts are coming to naught.

## THE CONTEXT OF RUSSIAN–GERMAN ECONOMIC RELATIONS AT THE BEGINNING OF 2022

After the start of Russia’s special military operation on the territory of Ukraine, German companies began to decide on the further strategy for their presence in the Russian market. Their activities, even in nonsanctioned sectors, were under massive reputational pressure generated by the media. Most large German companies announced their withdrawal from the Russian market or radically reduced the scope of their activities, leaving the possibility of selective cooperation under previously concluded contracts.

The main sphere—energy cooperation between the two countries—has not become a safety device for maintaining the achieved level of economic relations. Over the past 50 years, it has played a positive role in promoting detente in international relations [Bros, Mitrova, and Westphal, 2017]. Its influence was also reflected in the inclusion of Russia after the collapse of the Soviet Union in the world financial and economic structures, especially when Berlin in the 2000s set the tone for Europe’s relations with its “resurgent neighbor” [Rahr, 2007; Stelzenmueller, 2009]. N.P. Pavlov rightly noted that the very mechanism of “special relations” between countries in the economic sphere clearly went wrong [Pavlov, 2021]. Other researchers predicted that the process of geopolitical changes and the evolution of economic relations between Russia and Germany based on the consumption of Russia’s energy resources could not proceed without conflict [Gutierrez del Cid, 2018].

Although tensions between the two countries have been steadily growing since December 2021, it seemed to experts that after the pandemic downturn in 2020, trade and economic relations were generally gaining a new chance for sustainable growth [Belov, 2020]. In this regard, we note that the coronavirus did not destroy several key areas of cooperation conceptualized by domestic researchers: in hydrogen energy [Belov, 2020a], in the mining industry [Sergeev and Lebedeva, 2016; Pavlov, 2019], in the digitalization of industry and innovation [Belov, 2020; Tarasova, 2020; Posselt and Rauch, 2011], and in the further localization of industrial projects [Belov, 2020b; Fedorov, 2013].

The countries approached the aggravation of the crisis in Ukraine with a trade turnover of about €59.8 billion in 2021, which was 34.1% more than in the previous year (exports and imports of Germany, €26.6 and €33.1 billion, respectively).^1^ With a share of 2.3% in Germany’s foreign trade, Russia has become one of the 15 most important trading partners.^2^

As for the landscape of German business in the Russian Federation, according to the latest data from the Bundesbank, in 2019 German investors controlled 472 companies in Russia. They employed almost 129 000 people, and the annual sales amounted to €38.1 billion. This corresponds to a share of 1.5% of the global annual turnover that German investors’ companies achieved abroad.^3^ Note that even before the aggravation of the crisis in Ukraine in 2021, the number of firms from Germany in Russia, recorded according to the methodology of the Russian–German Foreign Chamber of Commerce (AHK), had decreased by 8% (to 3651 from 6300 in 2011).^4^

## FEATURES OF DECISION-MAKING BY GERMAN FIRMS IN LIGHT OF THE SANCTIONS POLICY

Germany, having taken the lead in formulating and promoting five packages of EU sanctions, unlike in 2014, did not actively try to combine the sanctions policy with a diplomatic approach to resolving the Ukrainian crisis. Previously, it had looked for ways to engage Moscow in broader security issues [Siddi, 2016] to promote democracy and construct a new “Ostpolitik” [Doctorow, 2016] and to deal pragmatically with the consequences of disagreements for the EU economy [Kholodilin and Netsunajev, 2018; Gröschl and Teti, 2021]. As part of the first EU sanctions against the Russian Federation (February 2022), export bans were imposed on a number of essential goods and technologies. Sanctions and other measures to restrict exports are primarily a demonstration of the position of Germany and the EU [Basov, 2016]. Statistics will show in the next few months how they will affect Germany’s foreign trade with the Russian Federation.^5^

As a result of the sanctions decisions, the choice of a further strategy for the presence of German companies in the Russian market clearly fits into the general Western reaction. According to *Yale Researchers*, as of mid-April 2022, about 600 large international companies had left Russia, and about 130 remained.^6^ The list is constantly updated by the staff of the Yale School of Management to reflect the latest changes.^7^

There is every reason to believe that the preservation of relations is not an attempt to take into account Moscow’s policy but rather the conservation of activities and the hope for gradual changes in Russia [Handl, 2019]. At the same time, there is an intention to gain a foothold in the Russian market in conditions when the situation with the business climate within the country needs to be seriously improved [Zaritskiy, 2020].

Russia is working out, within the framework of the draft law On External Administration for Managing an Organization,^8^ three possible scenarios for the development of events in connection with the statements of working foreign manufacturers about leaving Russia or suspending their activities on Russian territory: first, the company continues its full-fledged work; second option, foreign owners transfer their shares under the management of Russian partners and subsequently will be able to return to the Russian market; third, the enterprise stops working, production is closed, and employees leave. The Eastern Committee of the German Economy (OstAusschuss, OA) noted in a statement that the new model threatens companies if they stop their activities in the Russian Federation.^9^

Public statements of leading German companies are presented in the materials of their press services or representative offices in Russia. By the nature of the announcements, the decisions made can be classified according to the following options: exit from the market, contraction of activity, stabilization–continuation of work in a nonsanctioned (at least at the moment) framework. Companies that chose to stay are under increasing pressure, including from the German business community itself.

### Exit from the Market

Representatives of the first, most massive exit strategy are most of the large German companies representing the IT sector, automotive and machine tool building, financial and insurance sectors, and the aviation industry. Thus, the German developer of ERP systems SAP stopped sales in Russia and “paused” further negotiations. The company for five years has gone down from 49% of a share of the Russian ERP market to 11%.^10^

In the automotive sector, the *BMW Group* has suspended deliveries to the Russian market and local production of cars due to the current geopolitical situation. Service maintenance of customer cars is not limited yet in compliance with all company standards.^11^*Volkswagen,* one of the anchor residents of the Kaluga automotive cluster, has also put its business on pause. The cessation of production of the concern will take place in Nizhny Novgorod at the site of the GAZ group.^12^*Audi,* as part of the *VW* group, due to the difficult situation “by all indicators,” stopped shipping cars to Russian dealerships from February 24, 2022.^13^ The German tire manufacturer *Continental* has stopped business and production in Russia. The company had, like Volkswagen, a plant in Kaluga.^14^*Daimler Truck* stopped working with KamAZ. The company refused to produce trucks and supply components, although it had been cooperating with the Russian concern for 12 years. In addition, *Mercedes-Benz,* the subsidiary of which is *Daimler Truck,* is exploring the possibility of selling its stake in the Russian company (15%).^15^

In the banking sector, *Commerzbank* ceased operations in Russia. The same decision was made by one of the largest banks in Germany, *Deutsche Bank*, and it announced the closure of the remaining business. It does not intend to participate in new projects in the Russian Federation, the statement says.^16^ The German airline *Lufthansa* refrained from flying in Russian airspace until May 27. The reason for this decision was the “current regulatory situation.” The service division of *Lufthansa Systems* refused to cooperate with Russian customers.^17^*Allianz SE* insurance company has stopped insuring new businesses and will not make new investments.^18^

In the machine tool industry, at the end of February, *DMG MORI* ceased sales and service activities in Russia, as well as production in Ulyanovsk. This also includes the supply of machines, spare parts, components, and services. In total, about 200 employees of a modern production and assembly plant in Ulyanovsk, as well as three trading and service companies in Moscow, Ulyanovsk, and Yekaterinburg, were affected.^19^

### Contraction of Activity

Almost a month after the start of the special military operation, not all major international concerns had announced a temporary withdrawal from the market. Companies in the electrical, chemical, pharmaceutical, medical, resource, consumer, and logistics sectors have continued to operate and do not plan to close, while significantly reducing the scale of their activities.

*Siemens* announced the suspension of product deliveries to Russia. At the same time, the company promised to carry out maintenance and repair of equipment “in strict accordance with the sanctions.” On March 15, it became known that the concern will continue maintenance of the *Lastochka* and *Sapsan* trains in the Russian Federation.^20^ Agricultural products, medical equipment, and medicines were taken out of the scope of the sanctions. The medical division of *Siemens Healthineers*, one of the world’s largest manufacturers of medical equipment, remained in the market.^21^

The *Bosch* concern said it was studying the sanctions, including measures against individuals. The company’s customers have been warned about possible delays in deliveries from abroad. Supply disruptions are already being felt in the auto parts segment. *Bosch* experts are in close contact with Russian partners, but, as an international company headquartered in Germany, they are obliged to comply with the requirements of European law.^22^ The company has been forced to close its plant in St. Petersburg due to the EU ban on the import of components. *Bosch*, the main contractor of AvtoVAZ microelectronics, sent employees on vacation due to supply problems.^23^

In a special statement, *Bayer* Chemical and Pharmaceutical Concern recalled that it supplies Russia with medicines for the treatment of cancer and cardiovascular diseases, pharmaceutical products to maintain the health of pregnant women and children, and seeds for growing food. At the same time, the company has suspended “insignificant types of business” in the Russian Federation and Belarus for an indefinite period (the concern included new investment projects, marketing events, and the placement of any advertising^24^).

*Fresenius*, a manufacturer of medical equipment, has a similar argument: “Our responsibility as a healthcare company is also not to abandon our patients in Russia.” Since 2005, the company has operated more than 90 dialysis centers in 36 regions of the Russian Federation. In addition, about 300 clinics and research institutions are equipped with its equipment. It should be noted that the revenue from business in Russia at the parent concern *Fresenius* in 2021 was significantly less than 1% of the total turnover of €37.5 billion.

The pharmaceutical company *Stada* found itself in a more difficult situation. For this manufacturer of generics and OTC drugs, Russia was the second largest sales market after Germany, where one in six of its employees (approximately 2100 people) work. The share of the local market in the global revenue of approximately €3 billion exceeded 14%. *Stada* has 20 production sites around the world, of which two plants are in Russia: Nizhpharm in Nizhny Novgorod and Hemofarm in Obninsk. The company offers over 170 types of drugs in the Russian Federation, and the level of localization of production (the use of raw materials, components, and equipment of Russian origin) for the industry exceeds 65%.^25^

In the chemical industry, *Henkel*, having fully supported “all sanctions against the country, its government, and the financial sector,” suspended investments, and ceased advertising in state media and sponsored activities. At the same time, the concern announced that for the time being it intends to supply Russia with consumer goods but will carefully monitor the situation and make further decisions. About 2500 employees work at *Henkel* enterprises at 11 plants in Moscow, Leningrad, Saratov, Ulyanovsk, Chelyabinsk, and Novosibirsk oblasts, as well as in Stavropol’ and Perm’ krais. Until now, Russia has provided approximately 5% of the group’s annual revenue, which in 2021 amounted to over €20 billion.^26^

The restrictions will also affect the Arctic vector of cooperation between Russia and Germany [Kotov, 2020]. However, *Wintershall* will continue to participate in three gas projects with Gazprom. The company explained the preservation of its stake in existing projects in Russia by the fact that it is the production of natural gas for the energy supply of Europe.^27^

The German sports shoes, apparel, and accessories group *Adidas AG* has suspended its own stores and online store but has continued to pay salaries to employees for the time being.^28^ The transport company DHL has stopped accepting and delivering international parcels to Russia for an indefinite period; at the same time, the circulation of goods and documents in the domestic market is carried out in the normal mode.^29^

### Stabilization and Continuation of Work

In the category of those who announced the continuation of work in the Russian market in full, there are large companies in the consumer and construction sectors, using mainly domestic raw materials and goods. The decision to stay in Russia was made by two large German retail chains, *Metro* and *Globus*, which in the Russian Federation provided, respectively, almost 10 and 15% of global turnover. *Metro* Russia was going to continue to serve small and medium-sized businesses from the field of catering and retail trade. Over 10 000 employees work at the concern’s enterprises in Russia. *Globus* denied reports of leaving the Russian market.^30^ The key shareholders of the concern—the *Meridian* fund and the *Beisheim* holding—supported the decision of the board to continue work.^31^

One of the leaders in the building materials market, *Knauf*, was not planning to stop its work in the Russian market. “We will stay as long as the political and commercial situation allows us to do so. We are clearly responsible for more than 4000 employees and their families, as well as customers and suppliers, with many of whom we have had excellent and long-standing relationships over the years,” the company assured. Since 1993, the manufacturer has invested more than 1.65 billion euros in the Russian economy. At the beginning of 2022, the group of companies included 20 factories, six sales organizations, six training centers, and 29 resource centers in the secondary vocational education system as part of the corporate academy.^32^

## FAINT HOPE FOR A NEW BEGINNING

German entrepreneurs express concern about the possible introduction of external management in German companies that have suspended their activities. The German business community as a whole supported the anti-Russian sanctions. The situation is different from 2014. Now we are talking not about adapting to sanctions, but about maintaining, no matter how significant, the nature of trade and economic relations between Russia and Germany. German firms that remain in the market make their continued presence directly dependent on the completion of the special military operation in Ukraine.

This is the first time that such a situation has arisen, indicating a significant revision by German business circles of their attitude to their prospects in the Russian market. The German foreign economic policy towards the Russian Federation is eroding the foundation of pragmatic and purely market relations. From spring 2014 to March 2022, the German business community declared its readiness to bear the costs associated with the events around Ukraine. This point of view is now a thing of the past.

In the current conditions, only individual platforms will remain, where episodic dialogue can be conducted on areas that are of most interest to German partners, taking into account the adjustment of the economic policy of Germany as a whole (for example, in the energy sector). A small window of opportunity remains for companies that have decided to remain in the Russian market and operate under the restrictive measures imposed by the Russian government. Finding solutions for companies in the face of political pressure and the threat of losing the market will be difficult. At the same time, the economic authorities of the Russian Federation, apparently, will offer acceptable working conditions for those economic entities that will continue their activities and become a help for future Russian–German economic ties.

## Footnotes

1 Trade between Russia and Germany grew by 34.1% in 2021—trade representative of the Russian Federation. https://www.finam.ru/analysis/newsitem/tovaroobmen-mezhdu-rossieiy-i-germanieiy-v-2021-godu-vyros-na-34-1-torgpred-rf-20220214-204654/. Cited March 25, 2022.

2 Twelfth and 14th most important in terms of total imports and exports; 4th and 5th in imports and exports, respectively, among countries outside the EU.

3 Fakten zum Außenhandel mit Russland. https://www.destatis.de/DE/Presse/Pressemitteilungen/2022/02/PD22_N010_51.html. Cited March 20, 2022.

4 The website of the German–Russian Chamber of Commerce at the time of writing the note was closed for reconstruction with the termination of access to all previously published expert materials and reviews. Only emergency contacts worked (anticrisis hotline, communication with management, and representative office in Germany). https://russland.ahk.de/ru/vvedutsja-tekhnicheskie-raboty?ysclid=l0zgf9jh9s&cHash=3d57a1daa016 d33afbacab5cb813c506. Cited March 20, 2022.

5 Außenhandel mit Russland im Januar 2022 gegenüber dem Vorjahresmonat gestiegen. https://www.destatis.de/DE/Presse/Pressemitteilungen/2022/03/PD22_110_51.html. Cited March 20, 2022.

6 Not everyone leaves. The world’s largest chains that remain in Russia. https://www.malls.ru/rus/news/krupneyshie-mirovye-seti-kotorye-ostayutsya-v-rossii.shtml. Cited March 20, 2022.

7 Over 600 companies have withdrawn from Russia, but some remain. https://som.yale.edu/story/2022/over-600-companies-have-withdrawn-russia-some-remain. Cited April 14, 2022.

8 Submitted to the State Duma of the Federal Assembly of the Russian Federation on April 12, 2022.

9 Verantwortung für die Mitarbeiterinnen und Mitarbeiter. https://www.ost-ausschuss.de/de/verantwortung-fuer-die-mitarbeiterinnen-und-mitarbeiter. Cited March 20, 2022.

10 SAP stopped sales in Russia and “paused” negotiations. https://www.cnews.ru/news/top/2022-03-03_nemtsy_pokidayut_ rossiyu_iz-za. Cited March 20, 2022.

11 BMW leaves Russia. https://www.vedomosti.ru/auto/articles/ 2022/03/01/911583-bmw-uhodit-iz-rossii. Cited March 20, 2022.

12 VW-Konzern stellt Russland-Geschäft ein. https://www.tagesschau.de/wirtschaft/vw-russland-101.html. Cited March 20, 2022.

13 Importers began to refuse deliveries of cars to Russia. https://www.vedomosti.ru/business/articles/2022/02/24/910843-importeri-otkazivatsya-mashin. Cited March 20, 2022.

14 German tire manufacturer Continental suspends production and business in Russia. https://tass.ru/ekonomika/14001907?. Cited March 20, 2022.

15 Daimler Truck suspends work with KamAZ. https://www.rbc.ru/technology_and_media/28/02/2022/621c 99b59a79475a82032b79. Cited March 20, 2022.

16 Deutsche Bank leaves Russia. https://1prime.ru/banks/ 20220312/836350587.html. Cited March 20, 2022.

17 Friends among strangers: What will happen to the personnel of outgoing foreign companies in the Russian Federation. https://www.buhgalteria.ru/article/svoi-sredi-chuzhikh-chto-budet-s-personalom-ukhodyashchikh-inostrannykh-kompaniy-v-rf. Cited March 20, 2022.

18 Allianz suspends new business and investment insurance in Russia. https://ua.interfax.com.ua/news/general/813517.html. Cited March 20, 2022.

19 “‘About 200 employees were affected’: DMG MORI closed production in Ulyanovsk back in February,” Ural-Pressa, Mar. 15 (2022).

20 Plus ASUS and The North Face: List of companies that have stopped or suspended work in Russia. https://biz.nv.ua/markets/kakie-kompanii-uhodyat-iz-rossii-spisok-poslednie-novosti-50222290.html. Cited March 20, 2022.

21 “Everything will be more expensive, more complicated, and slower”: Arkady Stolpner on what will happen to Russian medicine. https://thebell.io/vse-budet-dorozhe-slozhnee-i-medlennee-arkadiy-stolpner–o-tom-chto-budet-s-rossiyskoy-meditsinoy. Cited March 20, 2022.

22 Which German companies remain in Russia? https://aussiedlerbote.de/2022/03/kakie-nemeckie-kompanii-ostayutsya-v-rossii/. Cited March 20, 2022.

23 List of companies that leave and stay in Russia. https://rozetked.me/cancellations/. Cited March 20, 2022.

24 Bayer suspends advertising and investments in Russia and Belarus. https://ria.ru/20220314/bayer-1778078942.html?. Cited March 20, 2022.

25 For money or for Russians? Which firms from Germany continue their business in the Russian Federation. https://www.facenews.ua/news/2022/522028/. Cited March 20, 2022.

26 Henkel continues operating and manufacturing activities in Russia. https://www.kommersant.ru/doc/5249206. Cited March 20, 2022.

27 Wintershall Dea remains in Russian upstream projects but writes off investments in Nord Stream 2. https://neftegaz.ru/news/companies/728245-wintershall-dea-ostaetsya-v-rossiyskikh-dobychnykh-proektakh-no-vlozheniya-v-severnyy-potok-2-spisyv/. Cited March 24, 2022.

28 Adidas will suspend the work of its stores in Russia. https://www.rbc.ru/business/08/03/2022/62266b5d9a7947d348fb389f. Cited March 20, 2022.

29 DHL: The company does not stop work in the Russian market. https://www.bfm.ru/news/494233. Cited March 20, 2022.

30 Auchan and Globus say they do not plan to leave the Russian market. https://www.kommersant.ru/doc/5251119. Cited March 20, 2022.

31 For money or for Russians? Which firms from Germany continue their business in the Russian Federation. https://www.facenews.ua/news/2022/522028/. Cited March 20, 2022.

32 Knauf will continue to work in Russia. https://realty.interfax.ru/ru/news/articles/134596/. Cited March 20, 2022.
